# A clinical decision support tool to screen health records for contraindications to stroke thrombolysis–a pilot study

**DOI:** 10.1186/s12911-015-0229-4

**Published:** 2015-12-14

**Authors:** Mu-Chien Sun, Jo-Ann Chan

**Affiliations:** Stroke Center and Department of Neurology, Changhua Christian Hospital, #135, Nanhsiao Street, Changhua, 500 Taiwan

**Keywords:** Biomedical technology, Brain infarction, Decision making, Computer-assisted, Decision support systems, Clinical, Hospital information systems, Medical records systems, Computerized, Medical informatics, Stroke, Thrombolytic therapy

## Abstract

**Background:**

The use of intravenous thrombolysis for stroke is limited by contraindications that may be difficult to identify promptly and accurately. Evidence supports the use of information technology-based clinical decision support (CDS) tools to achieve improvements in care delivery. The objective of this pilot study was to investigate the efficacy of a CDS tool to screen health records for contraindications to intravenous stroke thrombolysis.

**Methods:**

A CDS tool was developed to rapidly screen health information in seven affiliated hospitals for contraindications to stroke thrombolysis. A fixed-sequence, 2-period crossover study was conducted to test the efficacy of the CDS tool. Four mock patient records derived from the stroke registry that contained a total of nine contraindication items in two or more of the hospitals were used for testing purposes. The test patients were preset and balanced between groups with and without the CDS tool appearing six times in each group before recruiting the participating physicians. Physicians who were responsible for thrombolytic therapy and willing to sign informed consent were recruited. The participating physicians were asked to check a list of contraindications for two of the patients by using a shared electronic medical record system among the seven hospitals with and without the CDS tool. The test time and missed contraindications were recorded and analyzed statistically.

**Results:**

A total of 14 physicians who were responsible for stroke thrombolysis were approached, and 12 signed informed consent and took the test. By using the CDS tool, the test time was reduced significantly from 14.6 ± 7.4 to 7.3 ± 5.2 min (*P* = 0.010). In a total of 54 contraindications, the number of missed contraindications was reduced significantly from 23 (42.6 %) to seven (13.0 %) (*P* = 0.001). The difference of missed contraindication number between the two groups was statistically significant either per physician or per contraindication item.

**Conclusions:**

By screening health records for relevant contraindications, the use of a CDS tool may reduce the time needed to review medical records and reduce the number of missed contraindications for stroke thrombolysis.

**Electronic supplementary material:**

The online version of this article (doi:10.1186/s12911-015-0229-4) contains supplementary material, which is available to authorized users.

## Background

Strong evidence supports the use of clinical decision support (CDS) tools and computerized provider order entries [1]. A systematic review of the literature shows that most studies report that health information technology (IT) interventions had statistically and clinically significant benefits on health care [1].

Intravenous thrombolysis with tissue-type plasminogen activator (tPA) for acute ischemic stroke is recommended by stroke guidelines [2]. However, the beneficial effects of this treatment are highly time-dependent [3]. Because of the risk of hemorrhage, and especially intracerebral hemorrhage, the use of intravenous tPA thrombolysis is limited by certain criteria, especially contraindications [4]. Major deviations from protocol may place patients at a higher risk of in-hospital mortality, serious extracranial hemorrhage and symptomatic intracerebral hemorrhage [5, 6]. A detailed medical history of patients with regards to contraindications for treatment must be taken carefully and promptly from the patients themselves and/or family members to avoid complications. However, this may not always be possible when the patient is confused or aphasic. In these situations, health IT systems may provide an opportunity for safer, more efficient care.

Changhua Christian Hospital (CCH) is one of the major providers of intravenous thrombolysis treatment for stroke in Taiwan [7]. The safety and efficacy of treatment have been shown to be comparable to other Asian and Western countries [7, 8]. Changhua Christian Healthcare System (CCHS) is composed of CCH and six affiliated hospitals in the surrounding area in western Taiwan, covering a population of about 1.5 million people. CCHS developed a CDS tool, CCHS iStroke, to allow for the rapid screening of a relevant medical history for stroke patients requiring thrombolysis. The aim of this pilot study was to investigate the efficacy of this tool.

## Methods

### CCHS

Patients who visit CCHS hospitals for medical care are covered by the National Health Insurance program in Taiwan. Through this program, insured patients are free to visit any hospital at any time. Medical records at the affiliated hospitals can be accessed from CCH through a shared electronic medical record system (EMR) via a secured internet link. Patients who visit CCHS hospitals are encouraged to sign informed consent to allow for sharing health information among the CCHS hospitals, and treating physicians are authorized to obtain their medical records remotely from hospitals within CCHS if they have signed such consent.

### CCHS iStroke

**Fig. 1:**
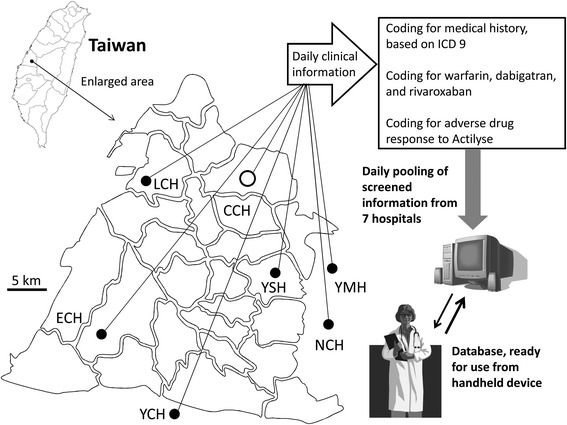
Changhua Christian Healthcare System iStroke system. CCH: Changhua Christian Hospital; ECH: Erlin Christian Hospital; ICD: International Classification of Diseases; LCH: Lukang Christian Hospital; NCH: Nanto Christian Hospital; YCH: Yunlin Christian Hospital;YMH: Yomin Hospital; YSH: Yuanshen Hospital . The authors own the copyright for the map image

**Fig. 2:**
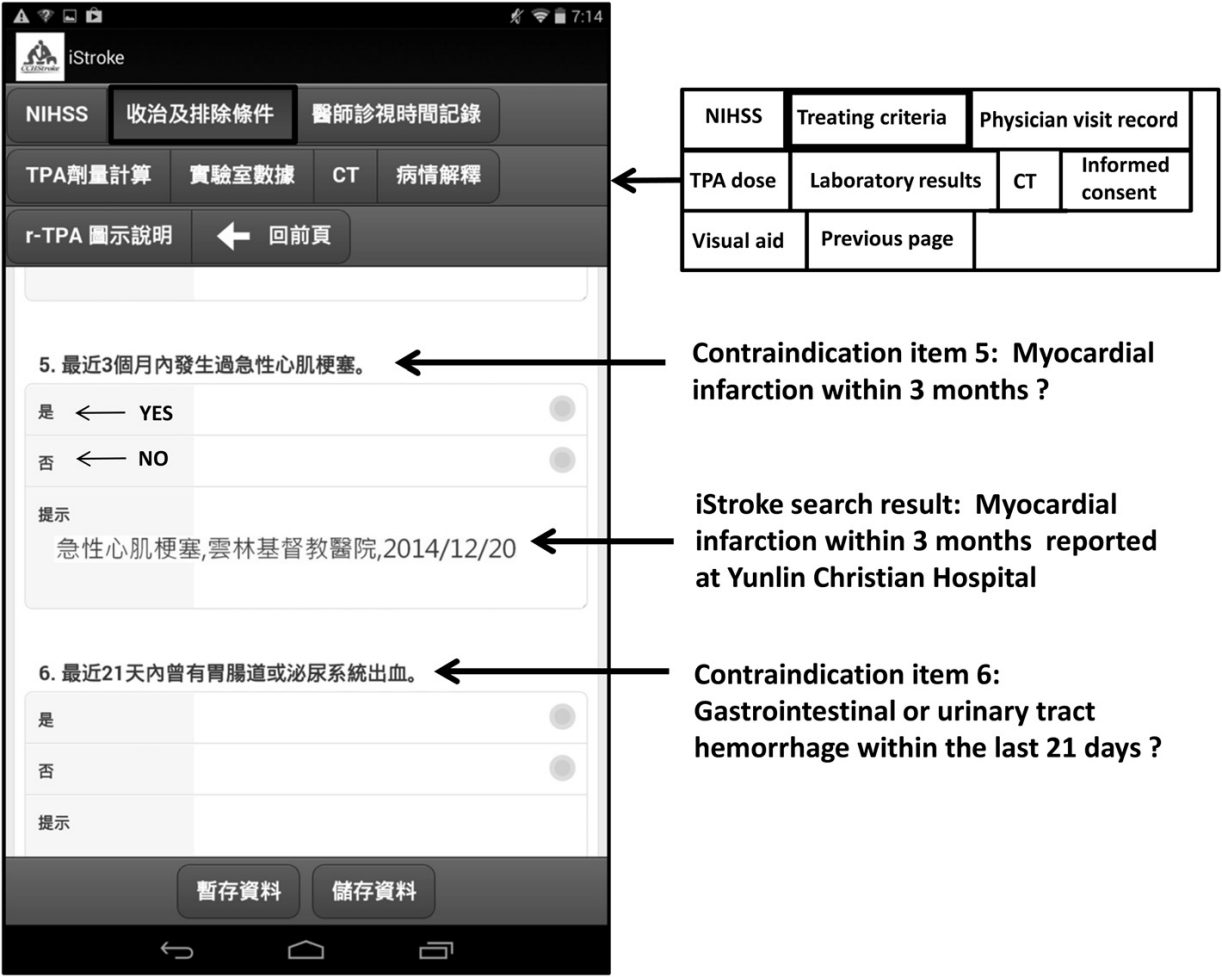
A screenshot of Changhua Christian Healthcare System iStroke. CT: computed tomography; NIHSS: National Institue of Health Stroke Scale; TPA: tissue-type plasminogen activator

There are a number of contraindications including medical history, medication use, and adverse drug responses for intravenous thrombolysis with tPA for acute ischemic stroke. In a total of 26 contraindications, 17 (65 %) are regarding information that can be extracted from medical records reported before the stroke. Contraindications that contain searchable items were identified, including adverse drug response records, anticoagulant use, and International Classification of Diseases codes in admission and out-patient records (Additional file 1: Table S1). The CCHS iStroke was developed to allow for rapid screening of the relevant medical history for stroke thrombolysis among all electronic CCHS hospital records to support clinical decision making. The CCHS iStroke is composed of two parts: a database and a handheld device with application software (app) that can access the database. The database is independent from the shared EMR and generated by searching contraindications from the shared EMR of all affiliated hospitals for every patient who has ever visited CCHS and signed informed consent. The database is updated on a daily basis. The app accesses the database for a specific patient profile when code stroke is activated for that patient. The app also synchronously accesses the shared EMR of the seven hospitals for contraindications, laboratory results, and brain imaging results that were generated in the last 24 h (Fig. 1). The app includes forms for National Institute of Health Stroke Scale score assessment, treating criteria assessment, physician visit time record, informed consent, tPA dose calculator, relevant laboratory results, computed tomography images of the brain, and a visual aid for risk communication (Fig. 2). The result of contraindication item search is presented below the checkboxes of the item when the physician assesses for treating criteria (Fig. 2). The treating physician could further confirm this information with the patient and/or family member(s) or by checking the EMR to be able to make a clinical decision for thrombolysis more rapidly and accurately. All clinical information generated in the app including total dose of tPA and results of National Institute of Health Stroke Scale score are recorded in the database and can be printed as needed.

### Evaluation of the CCHS iStroke

The Institutional Review Board Committee A at CCH approved this study for human subjects. Written informed consent was obtained from all participants. To limit situational variables and increase the significance, four mock patient records were generated. These records were derived from the stroke registry that contained records from two or more CCHS hospitals [7]. Relevant medical histories were found in the shared EMR in four of the seven hospitals. Some relevant medical histories were identified not because they excluded treatment, but because more attention was required for their consequences or related treatment. For example, a history of atrial fibrillation did not fulfill the exclusion criteria; however patients receiving anticoagulation therapy for atrial fibrillation may be excluded from treatment. A history of dementia was found in all test cases, however none fulfilled the exclusion criteria (advanced dementia). Table 1 shows the relevant medical histories and related contraindications in the four test cases. Of the 17 total contraindications for stroke thrombolysis that can be extracted from the EMR, the four cases had six different and a total of nine contraindications. Case 2 had a record of liver cirrhosis at Nantou Christian Hospital that met contraindication item 13. Case 2 also met contraindication item 15 (hemodialysis) as the risk of bleeding was considered to outweigh the benefits of therapy. Case 3 had a record of intracranial hemorrhage at CCH in 2010 that fulfilled contraindication item 1. Case 4 was taking anticoagulants from CCH and thus fulfilled contraindication item 16. Three of them met contraindication item 17 for a known history of diabetes and stroke.

**Table 1:** Relevant medical history and contraindication items fulfilled according to the test cases

Cases	Medical history (Hospital, Year)	Contraindication item
1	Dementia (YCH, 2007); Diabetes (YCH, 2009); Ischemic stroke (CCH, 2012); Stroke in 3 months (YCH, 2014)	3, 17
2	Atrial fibrillation (NCH, 2010); Dementia (NCH, 2010); Diabetes (NCH, 2010); Heart failure (NCH, 2008); Hemodialysis (CCH, 2000); Ischemic stroke (NCH, 2009); Liver cirrhosis (NCH, 2009)	13,15,17
3	Atrial fibrillation (CCH 2012); Dementia (ECH, 2012); Heart failure (ECH, 2011); Intracranial hemorrhage (CCH, 2010); Ischemic stroke (CCH, 2012)	1
4	Atrial fibrillation (YSH 2005); Dementia (CCH, 2014); Diabetes (CCH, 2011); Ischemic stroke (CCH, 2011); Rivaroxaban use (CCH 2014); Stroke in 3 months (CCH, 2014); Warfarin use (CCH 2014)	3, 16,17

CCH: Changhua Christian Hospital; ECH: Erlin Christian Hospital; NCH: Nanto Christian Hospital; YCH: Yunlin Christian Hospital

A pilot study using a fixed-sequence, 2-period crossover design was conducted to study the efficacy of the CDS tool. The test patients were preset and balanced between groups with and without the CDS tool so that each mock patient appeared six times in each group before recruiting the participating physicians. Physicians who were responsible for thrombolytic therapy at CCH and were willing to perform the test of the CCHS iStroke during off-duty hours were recruited and informed consent was obtained. The total number of participating physicians was set at 12 because that each mock patient appeared six times in both groups with and without the CDS tool.

**Fig. 3:**
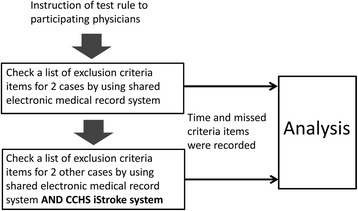
Study profile. CCHS: Changhua Christian Healthcare System

Each participant was presented with a set of stroke test cases. Each case began with the scenario of the patient arriving at CCH within 1 h of the stroke onset (Fig. 3). It was not possible to obtain any useful information regarding contraindications from the test case or his/her family members. For the control case, the participating physician was asked to check a list of contraindications for two test cases by using the shared EMR only (the control group). In the intervention case, the participant was asked to check a list of contraindications for another two test cases by using both shared EMR and CCH iStroke (the iStroke group). The time taken for the test and the number of missed contraindications were recorded for each set of test cases.

### Analysis

The missed contraindications were analyzed according to the participating physician. The time taken to review the medical records and the number of missed contraindications were compared statistically between the control and iStroke groups. Univariate analysis was carried out using unpaired t, Mann–Whitney, and χ square tests, whenever applicable. All statistically significant levels were defined as *P* < 0.05.

## Results

Every test case appeared six times in each group. There were a total of 54 contraindication items (6 × 9 items) in either the control or iStroke group. A total of 14 physicians who were responsible for thrombolytic therapy at CCH were approached consecutively, 12 of whom signed informed consent and were recruited for the test. Table 2 shows the characteristics of the 12 participating physicians. All of them had 3 or more years of clinical experience, and most had experience of intravenous thrombolysis with tPA for acute ischemic stroke in 10 or more patients.

**Table 2:** Characteristics of the participating physicians

Physician	Age, years	Gender	Clinical experience, years	Thrombolysis^a^ experience, case number	Test cases	
					Control	iStroke
#1	34	M	7	≧10	1,2	3,4
#2	38	M	13	≧10	1,3	2,4
#3	34	M	4	≧10	1,4	2,3
#4	30	M	4	≧10	2,3	1,4
#5	32	M	3	4-6	2,4	1,3
#6	30	M	3	≧10	3,4	1,2
#7	53	M	25	4-6	1,2	3,4
#8	43	M	14	≧10	1,3	2,4
#9	31	M	3	7-9	1,4	2,3
#10	43	M	18	≧10	2,3	1,4
#11	47	M	17	≦3	2,4	1,3
#12	31	F	5	≧10	3,4	1,2

^a^intravenous thrombolysis with tissue-type plasminogen activator for acute ischemic stroke

Table 3 shows a summary of the missed contraindications according to the participating physician. All participating physicians had at least one missed contraindication in the control group. Contraindication items 3, 13, and 17 were among the most frequently missed items. Notably, two physicians missed contraindication item one (previous intracranial bleeding at any time) and one missed item 16 (patient having received oral anticoagulants such as warfarin, dabigatran, or rivaroxaban) in the control group. Eight of the 12 physicians did not miss any contraindications in the iStroke group. Compared to control group, the number of missed contraindications was lower in the iStroke group in nine (75 %) of the participating physicians. Two had the same number of missed contraindications in both groups, and one had more missed items in the iStroke group.

**Table 3:** Missed contraindication items according to the participating physicians

Physician		Item #1	Item #3	Item #13	Item #15	Item #16	Item #17	c	i	Total
#1	c			1(2)			1(1)	2		2
	i								0	
#2	c	1(3)					1(1)	2		2
	i								0	
#3	c		1(4)					1		1
	i								0	
#4	c			1(2)				1		1
	i								0	
#5	c		1(4)	1(2)				2		2
	i								0	
#6	c		1(4)				1(4)	2		4
	i						2(1、2)		2	
#7	c			1(2)	1(2)			2		2
	i								0	
#8	c	1(3)						1		2
	i		1(4)						1	
#9	c		1(4)				2(1、4)	3		3
	i								0	
#10	c			1(2)	1(2)			2		3
	i						1(4)		1	
#11	c		1(4)			1(4)	1(4)	3		3
	i								0	
#12	c		1(4)				1(4)	2		5
	i			1(2)	1(2)		1(1)		3	
	c	2	6	5	2	1	7	23		
	i	0	1	1	1	0	4		7	
	Total	2	7	6	3	1	11			30

*c* control; *i* iStroke

Data shown as missed item count (test case number)

Table 4 shows the time taken for the test and the number of missed contraindications in the control and iStroke groups. The time was 14.6 ± 7.4 min in the control group and was only 7.3 ± 5.2 min in the iStroke group (*P* = 0.010). The total number of missed contraindication count was 23 in the control group and was only seven in the iStroke group (*P* = 0.001). The difference of missed contraindication count between the two groups was statistically significant either per physician or per contraindication item.

**Table 4:** Test results of control and iStroke groups

	Control	iStroke	*P*
Time, minutes, mean ± SD	14.6 ± 7.4	7.3 ± 5.2	0.010
Total missed contraindication counts, n (%^a^)	23 (42.6)	7 (13.0)	0.001
Missed contraindication counts per physician			
Mean ± SD	1.9 ± 0.7	0.6 ± 1.0	0.001
Median	2.0	0	0.002
Missed contraindication counts per item			
Mean ± SD	3.8 ± 2.5	1.2 ± 1.5	0.047
Median	3.5	1.0	0.027

*SD* standard deviation

^a^Missed item number in 54 (9 items × 6) contraindication items in each group

## Discussion

A recent review identified 26 currently available tools to support decision making or patient understanding in the treatment of acute ischemic stroke with intravenous thrombolysis [9]. Most of these tools were related to patient information and risk communication. Three of these were electronic tools that used predictive equations to calculate outcomes for individual patients. However, none of them were related to active screening of guideline or licensed contraindications that may be critical and time-consuming when making a decision for treatment [9]. Our results demonstrate the feasibility of a CDS tool of this kind.

Reports on the off-label use of tPA have shown that current guideline or licensed contraindications are subject to change [10]. A retrospective study analyzed protocol violations and rates of symptomatic intracerebral hemorrhage, and showed that protocol violations occurred in 36 % of enrolled patients. Nevertheless, there was no significant increase in the incidence of symptomatic intracerebral hemorrhage in these patients [11]. However, most common violations in the report were related to a 3 h-time window and blood pressure rather than other contraindications. Medico-legal issues are a concern in tPA thrombolysis for acute ischemic stroke. Although the rate of litigation claims involving complications from treatment was low in a recent review, fatal or poor outcomes may be a triggering factor for litigation [12, 13]. The incidence of symptomatic intracerebral hemorrhage in patients receiving intravenous thrombolysis for acute stroke has been reported to range from 2 to 6 %, and such cases are prone to legal litigation if a protocol violation has occurred [14]. Treatment following current guideline and licensed criteria may be the best choice, especially for clear-cut contraindications.

In our study, two physicians missed contraindication item 1 (previous intracranial bleeding at any time) and one missed contraindication item 16 (patient having received oral anticoagulants such as warfarin, dabigatran, or rivaroxaban) in the control group. However, none were missed in the iStroke group. Therefore, the CCHS iStroke may be a useful CDS tool to prevent medico-legal issues.

It has been demonstrated that small reductions in the delay of thrombolysis for stroke result in significant and robust average health benefits over the patients’ lifetime, with each minute of onset-to-treatment saved granting on average 1.8 days of extra healthy life [15]. Efforts should therefore be made to shorten the time required to make a decision. The use of the CCHS iStroke in our study resulted in a 50 % reduction in the time required to review medical records, and a 70 % reduction in the number of missed contraindications. The CCHS iStroke may therefore be an efficacy CDS tool to decide whether or not to initiate thrombolysis for stroke patients.

There are several limitations to this study. The use of mock patient records may be a better way to standardize the process and shorten the time required for testing. However, further studies in real world practice are required to investigate its effectiveness. Another limitation is that some contraindications for stroke thrombolysis were not included in our tool because of the requirement to measure them on site, including severe stroke with a National Institute of Health Stroke Scale score >25, platelet count ≤100,000/mm^3^, blood glucose level, and blood pressure. The CCHS iStroke was designed to support decision making rather than actually making the decision, and the final clinical decision is left to the treating physician. In addition, the screening protocol of the CCHS iStroke depends on searchable items defined by the hospital IT department and is thus subject to change. Our database includes patient records of one medical center (CCH) and six nearby community hospitals with a total of 3047 beds. It was an advantage to have all relevant medical records in the same healthcare system connected by a shared EMR, however this may not apply to hospitals in other areas. Nevertheless, the CCHS iStroke may be used within a single hospital.

## Conclusions

Our results of this pilot study demonstrate that the CCH iStroke may be an efficacy tool to support decision making for when to initiate intravenous thrombolysis for patients with acute ischemic stroke. Further studies in real world practice are required to investigate its clinical effectiveness.

## Additional file

Additional file 1: Table S1. Contraindications based on the Taiwan Stroke Society guideline. (DOC 36 kb)

## Abbreviations

CCH: Changhua Christian Hospital
CCHS: Changhua Christian Healthcare System
CDS: clinical decision support
IT: information technology
tPA: tissue-type plasminogen activator
