# Supplementary material for: A clinical decision support tool to screen health records for contraindications to stroke thrombolysis–a pilot study
Source: BMC Med Inform Decis Mak. 2015 Dec 14;15:105. doi: 10.1186/s12911-015-0229-4 (PMC4677442; doi:10.1186/s12911-015-0229-4)
Supplement: Additional file 1: Table S1. — Contraindications based on the Taiwan Stroke Society guideline. (DOC 36 kb) [file 12911_2015_229_MOESM1_ESM.doc]

**Supplementary Table.** Contraindications based on the Taiwan Stroke Society guideline.

| Item | Content |
| --- | --- |
| 1 | Previous intracranial bleeding at any time |
| 2 | Known allergy to Actilyse |
| 3 | Stroke within 3 months |
| 4 | Severe head trauma within 3 months |
| 5 | Myocardial infarction within 3 months |
| 6 | Gastrointestinal or urinary tract hemorrhage within the last 21 days |
| 7 | Major surgery or trauma within the last 14 days |
| 8 | Puncture of vessel at non-compressible site, such as subclavian or jugular vein, within the last 10 days |
| 9 | Known intracranial neoplasm or arteriovenous malformation |
| 10 | Hemorrhagic retinopathy, such as DM retinopathy |
| 11 | Infective endocarditis, pericarditis |
| 12 | Suspected aortic dissection |
| 13 | Advanced liver disease, such as hepatic failure, liver cirrhosis, portal hypertension, and acute hepatitis |
| 14 | Acute pancreatitis |
| 15 | Conditions that risks of bleeding are considered to outweigh the benefits of therapy, such as active pulmonary tuberculosis, hemodialysis, advanced heart failure, advanced dementia |
| 16 | Patient having received oral anticoagulant, such as warfarin, dabigatran, or rivaroxaban |
| 17 | Known history of diabetes and stroke |
